# Safety and efficacy of an interleukin 12/23 inhibitor in a patient with constitutional neutropenia and psoriasis vulgaris^☆^^☆☆^

**DOI:** 10.1016/j.abd.2020.11.010

**Published:** 2021-09-17

**Authors:** Jéssica Vianna Starek, Cristina Santos Ribeiro Bechara, Mariana Reis e Rocha Dultra, Juliana de Morais Fernandes Krakheche

**Affiliations:** a Complexo Hospitalar Padre Bento de Guarulhos, Guarulhos, SP, Brazil; b General Dermatology and Severe Psoriasis Outpatient Clinic, Complexo Hospitalar Padre Bento de Guarulhos, Guarulhos, SP, Brazil

Dear Editor,

Psoriasis is a chronic, immune-mediated and complex inflammatory disease. The immunopathogenesis of the disease involves interferon-gamma (IFN-gamma), tumor necrosis factor (TNF), and specific interleukins (ILs) that coordinate the interaction between inflammatory cells and keratinocytes.^1^

IL inhibitors represent a new group of biological agents with greater specificity for the treatment of psoriasis, as they selectively target inflammatory pathways.^1^

Ustekinumab is a fully human monoclonal antibody that binds with high affinity and specificity to the p40 protein subunit, shared by cytokines IL-12 and IL-23.^2^, ^3^ Its action prevents the binding of IL-12 and IL-23 to their receptor, blocking the Th1 and Th17-mediated inflammatory pathways.^3^, ^4^

Benign constitutional neutropenia is an asymptomatic condition characterized by mild chronic neutropenia (neutrophil count < 1500/mm^3^) in patients with no history of recurrent infections and no secondary causes.^5^ As these patients are susceptible to infections, the use of immunobiological agents in this population may require special care regarding their safety. There are no reports in the literature on the use and safety of IL-12 and IL-23 inhibitors in these patients.

A 44-year-old dark-skinned male patient started follow-up at a dermatology referral service 10 years ago due to severe psoriasis, without joint involvement. He had a previous diagnosis of familial constitutional leukopenia 17 years ago, with a mean leukocyte count of 2600 mm^3^ and neutrophil count of 770 mm^3^. He had been previously treated with acitretin, topical medications and undergone around 400 phototherapy sessions, but persisted with erythematous-desquamative plaques on the lower limbs, upper limbs, trunk, and scalp, with progressive worsening. In 2017, due to treatment refractoriness, with a Dermatological Life Quality Index (DLQI) of 13, Psoriasis Area and Severity Index (PASI) of 12.9 (Fig. 1) and, considering the constitutional neutropenia, the use of ustekinumab was proposed with the caveat of undergoing monthly monitoring and withdrawing the treatment if the patient had a neutrophil count < 500/mm^3^.

**Figure 1:**
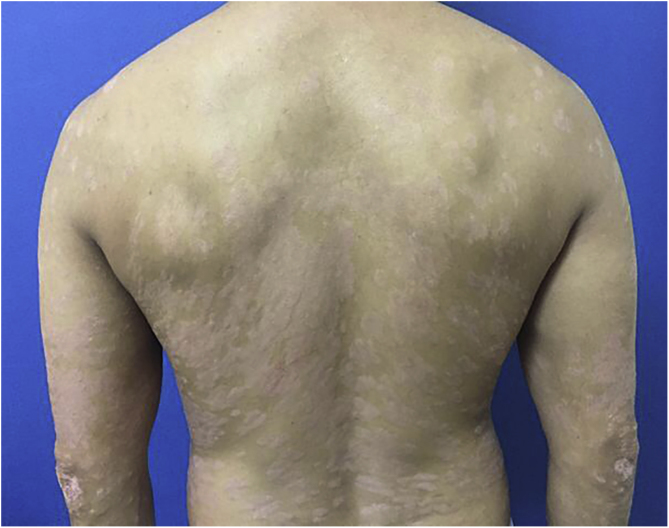
PASI 12.9, erythematous-scaling plaques.

After undergoing the initial tests before starting the immunobiological treatment, which were all within the normal range, treatment with ustekinumab 45 mg was introduced, with the induction phase taking place in weeks 0 and 4 and then every 12 weeks, associated with calcipotriol, twice a day. The patient showed significant lesion improvement after12 weeks, with residual macules only and currently with PASI 0 (Fig. 2). The patient continues to use the medication up to the present date, without any adverse effects or infections during the entire period (3 years of use). He maintained stable absolute neutrophil values, demonstrating the safety of the medication.

**Figure 2:**
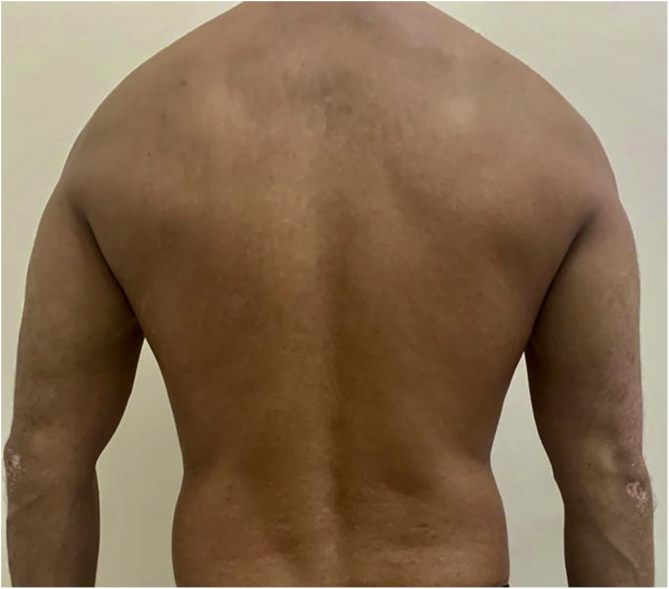
PASI 0, discrete residual macules after 3 years of treatment.

Evidence indicates that neutropenia during therapy with ustekinumab is rare, and when it occurs it is mild; therefore, periodic monitoring is recommended.^4^ As our patient already had a diagnosis of constitutional neutropenia, this adverse effect was the most feared one and the laboratory follow-up was performed monthly, and no relevant alterations were noted after 3 years of drug use.

## Financial support

None declared.

## Authors’ contributions

Jessica Vianna Starek: Approval of the final version of the manuscript; design and planning of the study; drafting and editing of the manuscript; collection, analysis and interpretation of data; critical review of the literature; critical review of the manuscript.

Mariana Reis and Rocha Dultra: Approval of the final version of the manuscript; drafting and editing of the manuscript; collection, analysis, and interpretation of data; critical review of the literature; critical review of the manuscript.

Cristina Santos Ribeiro Bechara: Approval of the final version of the manuscript; design and planning of the study; drafting and editing of the manuscript; collection, analysis, and interpretation of data; critical review of the manuscript.

Juliana de Morais Fernandes Krakheche: Approval of the final version of the manuscript; design and planning of the study; collection, analysis, and interpretation of data; effective participation in research orientation; intellectual participation in propaedeutic and/or therapeutic conduct of the studied cases; critical review of the literature; critical review of the manuscript.

## Conflicts of interest

None declared.
